# A Scoping Review of the Methodologies and Reporting Standards in Recent Applications of Artificial Intelligence in Radiomics for Chronic Subdural Hematoma Imaging

**DOI:** 10.7759/cureus.79163

**Published:** 2025-02-17

**Authors:** Nikhil Kota, Anusha Keshireddy, Anika Pruthi, Zain Abidin, Manisha Koneru

**Affiliations:** 1 Neurosciences, Cooper Medical School of Rowan University, Camden, USA; 2 Neurointerventional Surgery, Cooper University Health Care, Camden, USA

**Keywords:** ai, cerebrovascular disease, chronic subdural hematoma, deep learning, image analysis, machine learning, radiomics

## Abstract

Chronic subdural hematoma (cSDH) is the accumulation of blood in the subdural space, primarily affecting older adults. Radiomics is a rapidly emerging field that integrates artificial intelligence (AI) with imaging to improve diagnostic precision and prognostic predictions, including hematoma expansion and recurrence. However, the heterogeneous study designs, endpoints, and reporting standards limit its clinical application. This scoping review queried PubMed for studies published before or on December 25, 2024, using terms related to cSDH and AI-based imaging analysis. Inclusion criteria required primary research applying AI to cSDH imaging and reporting prognostic endpoints such as recurrence, expansion, or treatment response. Extracted data included methodological variables, imaging modalities, endpoints of interest, and performance metrics. Most studies used computed tomography (CT) imaging for analysis, with hematoma recurrence being the most frequently evaluated endpoint of interest. However, there was wide inconsistency in the reporting of model performance metrics. Thus, radiomics offers opportunities to improve outcome prediction and treatment planning in cSDH. Future work should focus on defining clinically meaningful endpoints, standardizing metrics, and validating models prospectively to facilitate integration into practice.

## Introduction and background

Chronic subdural hematoma (cSDH) is the accumulation of blood products within the subdural space, commonly affecting older adults and presenting with a wide range of symptoms, from asymptomatic cases to severe complications, such as herniation [1]. While prior approaches have included medical management (i.e., corticosteroids, tranexamic acid, statins), evidence supporting their efficacy has been inconsistent [2,3]. Surgical approaches, such as burr-hole drainage, craniotomy, or subdural evacuating port systems (SEPS), remain the mainstay of treatment and are associated with high success rates, though they carry risks of recurrence [4]. Recently, minimally invasive techniques, such as middle meningeal artery embolization (MMAE), have gained attention as a promising alternative with favorable outcomes [5]. The initial evaluation of cSDH relies on computed tomography (CT) imaging to assess hematoma size, expansion, and midline shift, and treatment decisions are dependent on radiographic and clinical evaluation [6].

Radiomics is a rapidly emerging field involving the extraction of quantitative features from medical imaging to produce analyzable data [7]. Advances in radiomics demonstrate the potential to enhance diagnosis and prognostic precision in cSDH. Artificial intelligence (AI)-based radiomics leverage automated or semi-automated imaging analytical methods to predict outcomes, such as hematoma expansion, recurrence, and patient prognosis [8,9]. However, the design, development, performance characterization, and reporting standards of these AI-based analytical models are highly heterogeneous. Variability in the prognostic endpoints of interest further limits the scope of the clinical application of radiomics-based models in routine practice [9]. This review aims to summarize recent work in the field of AI-based radiomics for cSDH imaging to provide insights into current reporting practices and highlight areas for future development.

## Review

Methods

This review was reported in accordance with Preferred Reporting Items for Systematic Reviews and Meta-Analyses (PRISMA) guidelines. This scoping review was conducted by querying PubMed published before or on December 25, 2024, with the following query: “(cSDH OR chronic subdural hematoma OR subdural hematoma OR subdural intracranial hematoma) AND (machine learning OR artificial intelligence OR deep learning OR radiomics)”.

Article titles and abstracts were screened by two independent reviewers based on inclusion and exclusion criteria. Studies must have not been a secondary analysis (i.e., commentary, review, conference proceedings, etc.), focused exclusively on cSDHs, and applied an AI method to imaging analysis.

The full-text review was performed by a single reviewer to extract the following methodological variables: total number of patient scans included, imaging modality (i.e., CT, magnetic resonance imaging (MRI), or digital subtraction angiography (DSA)), imaging analysis approach (i.e., deep learning or machine learning), and manual versus automated feature extraction. Additionally, the endpoints of interest for the included studies were collected, including hematoma recurrence, hematoma expansion, automated measurement, treatment response, hospital readmission, mortality, and clinical symptoms. Performance metrics of models were collected, including area under the curve (AUC), accuracy, sensitivity, specificity, and precision. The primary outcome is the proportion of methodological features and reported elements across included studies. Extracted data were summarized descriptively as medians with interquartile ranges (IQR) and frequencies using JMP version 18.0 (JMP Statistical Discovery LLC, Cary, NC) and Microsoft Excel version 16.78.3 (Microsoft Corp, Redmond, WA).

Results

**Figure 1:**
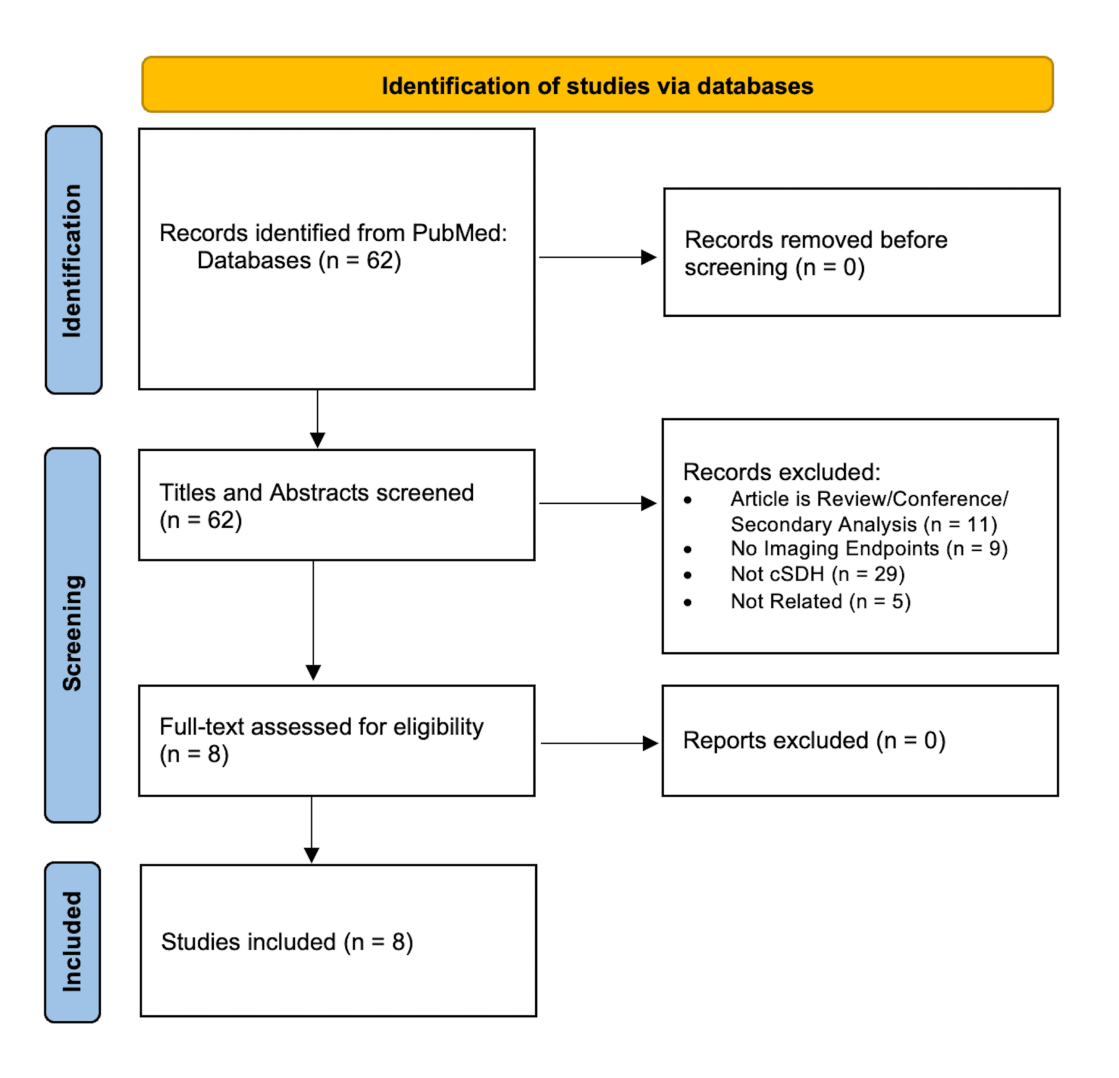
A PRISMA flowchart outlining the study selection process PRISMA: Preferred Reporting Items for Systematic Reviews and Meta-Analyses; cSDH: chronic subdural hematoma

Eight studies met inclusion criteria (Figure 1) [10-17]. Most studies involved analysis of more than 100 patient scans (75%, 6/8), and used CT as the primary imaging modality (100%) (Table 1).

**Table 1:** Included studies CT: computed tomography; MRI: magnetic resonance imaging

Study name	Total number of patients	Imaging modality
Fang et al. (2024) [10]	133	CT
Wu et al. (2024) [11]	64	CT
Zanaty et al. (2020) [12]	596	CT
Petrov et al. (2024) [13]	21	CT
Kellogg et al. (2021) [14]	128	CT
Inomata et al. (2024) [15]	215	CT
Ni et al. (2024) [16]	447	CT, MRI
Vargas et al. (2024) [17]	538	CT

**Figure 2:**
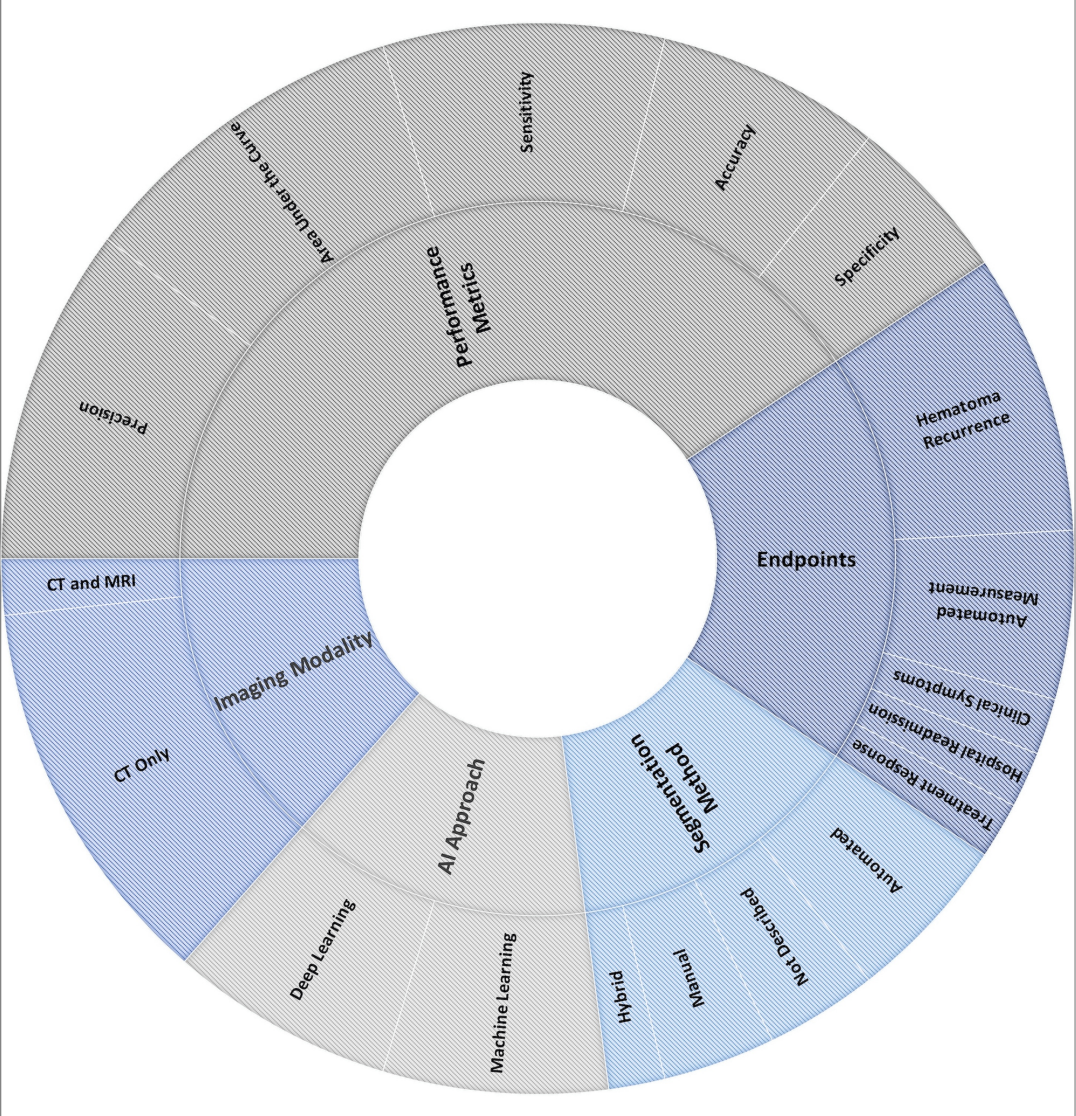
Sunburst diagram demonstrating relative frequency of design elements within artificial intelligence-based radiomics for chronic subdural hematoma imaging AI: artificial intelligence; CT: computed tomography; MRI: magnetic resonance imaging This figure has been created by the authors using Microsoft Excel version 16.78.3 (Microsoft Corp, Redmond, WA), derived from the analyses performed in the paper.

Approximately half of the studies used deep learning approaches as the primary AI-based image analysis strategy, while the other half of the studies used machine learning techniques (Table 2; Figure 2). For image processing and segmentation, there was generally proportional variation in reported use of automated, manual, and hybrid (e.g., semi-automated, mixed methods) approaches, although 25% of studies did not report their approach to image segmentation. Most studies used were developing AI-based image models to predict hematoma recurrence (62.5%, 6/8); approximately one-third of these studies were aimed to yield automated measurements of key imaging findings (i.e., hematoma volume, thickness, midline shift). A low proportion of studies attempted to prognosticate treatment response (12.5%), likelihood of readmission (12.5%), or clinical symptoms (12.5%). The reporting of model performance metrics was heterogeneous across studies. The most common metrics reported include AUC and model precision (75%). Specificity was only reported in approximately one-third of studies (Table 2).

**Table 2:** Summary of design and endpoint elements in included studies IQR: interquartile range; CT: computed tomography; MRI: magnetic resonance imaging; DSA: digital subtraction angiography

Characteristic	n = 8 studies
Number of patients, median (IQR)	174 (80-515)
Specified number included in training/validation sets, no. (%)	3 (37.5%)
Imaging modality, no. (%)	
CT only	7 (87.5%)
CT and MRI	1 (12.5%)
DSA	0 (0%)
Artificial intelligence approach, no. (%)	
Deep learning	4 (50.0%)
Machine learning	4 (50.0%)
Image segmentation method, no. (%)	
Automated	3 (37.5%)
Manual	2 (25.0%)
Hybrid	1 (12.5%)
Not described	2 (25.0%)
Model endpoint of interest, no. (%)	
Hematoma recurrence	5 (62.5%)
Automated measurement	3 (37.5%)
Treatment response	1 (12.5%)
Hospital readmission	1 (12.5%)
Clinical symptoms	1 (12.5%)
Progression/Expansion	0 (0%)
Mortality	0 (0%)
Reported performance metrics, no. (%)	
Area under the curve	6 (75.0%)
Accuracy	4 (50.0%)
Precision	6 (75.0%)
Sensitivity	5 (62.5%)
Specificity	3 (37.5%)

Discussion

Thus, we summarized common design and reporting practices for AI-based image analysis research in cSDH. Most studies included large numbers of scans, demonstrating a positive direction toward developing robust analysis algorithms based on large training sets. Notably, most of the studies were based on data extracted from CT; consequently, there is an opportunity to explore AI-driven models utilizing other imaging modalities. Although CT is more often obtained in routine cSDH clinical practice, the rise in MMAE also signifies more patients undergoing DSAs. Thus, future studies may analyze DSA data for developing imaging-based prognostic models, inclusive of real-time intra-procedural guidance, estimations of intraprocedural complications, and prognostication of post-MMAE recovery courses.

The most common clinical endpoint of interest across included studies was either estimating the likelihood of or quantifying the volume of hematoma recurrence. This aligns with the widespread use of hematoma recurrence as a primary or secondary endpoint in recent cSDH trials evaluating surgical and endovascular treatment, including the SQUID Trial for the Embolization of the Middle Meningeal Artery for the Treatment of Chronic Subdural Hematoma (STEM, NCT04410146) [18]; Embolization of the Middle Meningeal Artery with ONYX Liquid Embolic System for Subacute and Chronic Subdural Hematoma (EMBOLISE, NCT04402632) [19]; Managing Non-Acute Subdural Hematoma Using Liquid Materials: A Chinese Randomized Trial of MMA Treatment (MAGIC-MT, NCT04700345) [20]; Middle Meningeal Artery Embolization for the Treatment of Subdural Hematomas with TRUFILL n-BCA (MEMBRANE, NCT04816591) [21]; Chronic Subdural Hematoma Treatment with Embolization Versus Surgery Study (CHESS, NCT06347796) [22]; and Chronic Subdural Hematoma Treatment with Intra-Arterial Bevacizumab Injection (CHAI, NCT06510582) [23]. Given that several cSDH treatment trials assessed hematoma recurrence as a critical endpoint and that AI-based image analysis tools also examine this endpoint, there is potential scope for integrating these image analysis tools in cSDH trial workflows.

Future directions

However, there are gaps in current efforts for image-based methods of modeling and prognosticating other potentially useful endpoints for cSDH, such as aiding intra-procedural navigation for endovascular treatments and predicting the likelihood of complete resolution following treatment response. For instance, membranes within the hematoma collection observed on cSDH scans may indicate a higher degree of responsiveness to MMAE or novel anti-vascular endothelial growth factor agents [24-26]. Conversely, assessing the likelihood of clinical deterioration or mortality from cSDH could help guide informed decision-making, particularly in avoiding futile interventions and prioritizing quality of life when choosing treatment options. Future studies may focus on these endpoints to improve imaging model design for applications in clinical outcomes and decision-making.

Additionally, there is a lack of consensus on how the performance of imaging models is reported. To facilitate integration into clinical trials and practice, future research should focus on standardizing performance metrics and improving consistency in reporting. More comprehensive reporting of performance metrics is needed to assess model reliability, with a particular emphasis on rigorous validation in prospective clinical cohorts.

## Conclusions

We outlined common design and outcome elements in AI-based radiomics analyses for interpreting cSDH imaging. Future directions should focus on defining clinically meaningful endpoints to guide future applications of radiomics in cSDH. Moreover, future AI-based image analysis research should continue to adapt to clinical and technological advances in cSDH treatment, including new treatment approaches and imaging modalities. In light of recent clinical trials on surgical and endovascular treatments, standardizing performance metric reporting of these radiomics models will ensure consistency and reliability. Standardization will augment the integration of AI-based image analysis tools in both clinical trials and real-world workflows.
